# Update on Vaccine-Derived Polioviruses — Worldwide, January 2017–June 2018

**DOI:** 10.15585/mmwr.mm6742a5

**Published:** 2018-10-26

**Authors:** Jaume Jorba, Ousmane M. Diop, Jane Iber, Elizabeth Henderson, Kun Zhao, Roland W. Sutter, Steven G.F. Wassilak, Cara C. Burns

**Affiliations:** ^1^Division of Viral Diseases, National Center for Immunization and Respiratory Diseases, CDC; ^2^Department of Polio Eradication, Detection and Interruption Unit, World Health Organization, Geneva, Switzerland; ^3^Department of Polio Eradication, Research, Policy and Containment Unit, World Health Organization, Geneva, Switzerland; ^4^Global Immunization Division, Center for Global Health, CDC.

Since the Global Polio Eradication Initiative was launched in 1988 (*1*), the number of polio cases worldwide has declined by >99.99%. Among the three wild poliovirus (WPV) serotypes, only type 1 (WPV1) has been detected since 2012. This decline is attributable primarily to use of the live, attenuated oral poliovirus vaccine (OPV) in national routine immunization schedules and mass vaccination campaigns. The success and safety record of OPV use is offset by the rare emergence of genetically divergent vaccine-derived polioviruses (VDPVs), whose genetic drift from the parental OPV strains indicates prolonged replication or circulation (*2*). Circulating VDPVs (cVDPVs) can emerge in areas with low immunization coverage and can cause outbreaks of paralytic polio. In addition, immunodeficiency-associated VDPVs (iVDPVs) can emerge in persons with primary immunodeficiencies and can replicate and be excreted for years. This report presents data on VDPVs detected during January 2017–June 2018 and updates previous VDPV summaries (*3*). During this reporting period, new cVDPV outbreaks were detected in five countries. Fourteen newly identified persons in nine countries were found to excrete iVDPVs. Ambiguous VDPVs (aVDPVs), isolates that cannot be classified definitively, were found among immunocompetent persons and environmental samples in seven countries.

Global eradication of type 2 WPV (WPV2) was declared in 2015; type 3 WPV (WPV3) was last detected in 2012. The number of detected WPV1 cases has reached a historic low (22 cases in 2017 and 18 as of September 2018) in two of the three countries with endemic WPV1 transmission (Afghanistan and Pakistan); in Nigeria, WPV1 was last detected in September 2016. After the emergence of multiple cVDPV2 outbreaks during the preceding 15 years, in April 2016, all OPV-using countries switched from using trivalent OPV (tOPV; Sabin types 1, 2, and 3) to bivalent OPV (bOPV; Sabin types 1 and 3). To control and prevent cVDPV2 outbreaks, approximately 100 million doses of monovalent type 2 OPV (mOPV2) have been distributed in 11 countries (*4*). To maintain protection from poliovirus type 2 paralysis, 176 OPV-using countries have introduced at least 1 dose of injectable inactivated polio vaccine beginning in 2015.

## Properties and Virologic Characterization of VDPVs

Poliovirus isolates are characterized by laboratories of the Global Polio Laboratory Network. VDPV screening is conducted using real-time reverse transcription–polymerase chain reaction nucleic acid amplification, followed by sequencing of the VP1 region. VDPVs are isolates that are >1% divergent (for PV1 and PV3) or >0.6% divergent (for PV2) in VP1 nucleotide sequences from the corresponding OPV strain (*3*). Starting August 1, 2016, use of the VDPV2 screening assay was discontinued, and all PV2 isolates have been sequenced. VDPVs are further classified as 1) cVDPVs, when evidence of person-to-person transmission in the community exists; 2) iVDPVs, when they are isolated from persons with primary immunodeficiencies; and 3) aVDPVs, when they are clinical isolates from persons with no known immunodeficiency and no evidence of transmission, or they are sewage isolates that are unrelated to other known VDPVs and whose source is unknown (*2*).

## Detection of cVDPVs

**FIGURE 1:**
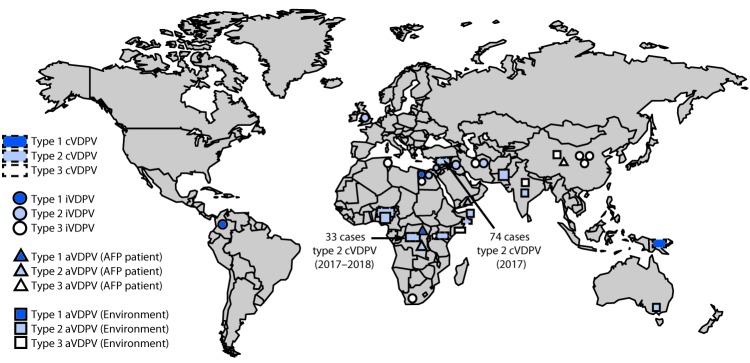
Vaccine-derived polioviruses (VDPVs) detected — worldwide, January 2017–June 2018 **Abbreviations:** AFP = acute flaccid paralysis; aVDPV = ambiguous VDPV; cVDPV = circulating VDPV; iVDPV = immunodeficiency-associated VDPV.

**FIGURE 2:**
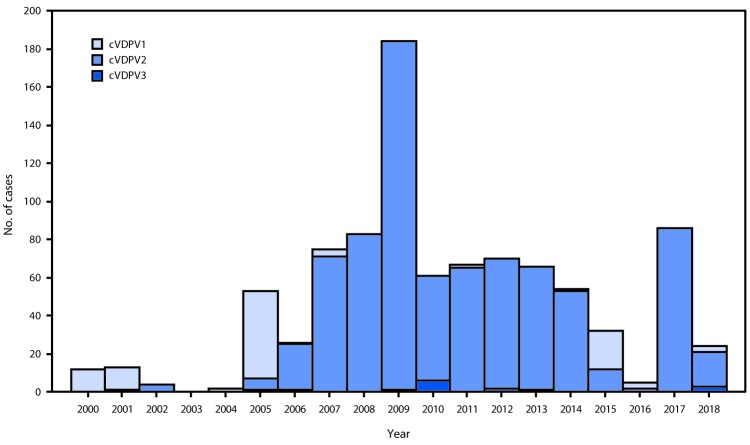
Circulating vaccine-derived poliovirus (cVDPV) cases detected, by serotype (N = 917) — worldwide, January 2000–June 2018* * Data through June 2018; available by September 18, 2018.

During January 2017–June 2018, cVDPV circulation was detected in six countries, an increase of one since the previous reporting period (*3*) (Figure 1); five countries reported cVDPV2 circulation (Democratic Republic of the Congo [DRC], Kenya, Nigeria, Somalia, and Syria); cVDPV1 was reported in Papua New Guinea (Table). Cases of cVDPV (in patients with acute flaccid paralysis [AFP]) continued to be identified from the previously reported (*3*) cVDPV2 outbreaks in Syria and DRC (*5*,*6*). No additional cases were reported from the cVDPV2 outbreaks first reported in Nigeria and Pakistan in 2016. New outbreaks were reported in DRC (three cVDPV2 emergences); Somalia (one cVDPV2 emergence and one cVDPV3 emergence) with linked cVPDV2 isolation in sewage in Kenya (*7*); Nigeria (two cVDPV2 emergences); and Papua New Guinea (one cVDPV1 emergence) (Table). During the reporting period, 113 cVDPV2 cases were detected (Table), including 74 in Syria, 33 in DRC, four in Nigeria, and two in Somalia. The large cVDPV2 outbreak in Syria (*3*) was apparently interrupted in 2017; the last reported cases had paralysis onset in September 2017. Three cVDPV1 cases were detected in Papua New Guinea, and three cVDPV3 cases were detected in Somalia (Table). Sixty-two cVDPVs were isolated from sewage sampling in environmental surveillance sites in Kenya, Nigeria, and Somalia. After June 2018, newly identified VDPVs linked to emergences during the reporting period were detected in all outbreak countries except Syria. During January 2017–June 2018, among 119 cVDPV cases, 113 (95%) were cVDPV2, which represented a serotype profile similar to that of the previous 12 years (Figure 2).

**TABLE:** Vaccine-derived polioviruses (VDPVs) detected, by classification and other selected characteristics — worldwide, January 2017–June 2018

Category	Country	Year(s) detected*	Source^†^	Serotype	Source of isolates^§^ January 2017–June 2018 (no.)			Capsid protein VP1 divergence from Sabin OPV strain** (%)	Coverage with 3 OPV doses (%)^††^	Estimated duration of VDPV replication^§§^ (yrs)	Date of last outbreak case, patient isolate, or environmental sample
					AFP cases	Non-AFP cases^¶^	Environmental surveillance				
**cVDPV**	DRC	2017–2018	Outbreak HLO-1	2	27	10	0	1.5–3.2	79	2.9	May 27, 2018
	DRC	2017	Outbreak MAN-1	2	2	1	0	0.7–1.0	79	0.9	Apr 18, 2017
	DRC	2018	Outbreak MON-1	2	4	3	0	2.1–2.4	79	2.2	Jun 24, 2018
	Kenya	2018	Outbreak BAN-1	2	0	0	2	4.9–5.2	81	4.7	May 29, 2018
	Nigeria	2018	Outbreak JIS-1	2	4	2	18	1.4–2.5	40	2.3	Jun 29, 2018
	Nigeria	2018	Outbreak SOS-3	2	0	0	17	0.7–1.2	40	1.3	Jun 26, 2018
	Papua New Guinea	2018	Outbreak	1	3	2	0	1.4–2.5	60	2.0	Jun 25, 2018
	Somalia	2017–2018	Outbreak BAN-1	2	2	0	16	3.7–4.8	47	4.7	May 29, 2018
	Somalia	2018	Outbreak BAN-2	3	3	1	9	1.6–2.1	47	1.9	May 18, 2018
	Syria	2017	Outbreak	2	74	60	0	2.4–3.7	53	3.3	Sep 21, 2017
**Total cVDPV**	**—^¶¶^**	**—^¶¶^**	**—^¶¶^**	**—^¶¶^**	**119**	**79**	**62**	**—^¶¶^**	**—^¶¶^**	**—^¶¶^**	**—^¶¶^**
**iVDPV**	China	2017	AFP patient	3	1	0	0	1.4	99	1.2	Oct 15, 2017
	China	2018	AFP patient	3	1	0	0	1.1	99	1.0	May 10, 2018
	China	2018	AFP patient	3	1	0	0	1.3	99	1.1	Jun 8, 2018
	Colombia	2018	AFP patient	1	1	0	0	1.4	92	1.2	May 16, 2018
	Egypt	2017	Non-AFP SCID	1	0	1	0	2.4	94	2.1	Oct 23, 2017
	Egypt	2017	AFP patient	2	1	0	0	1.9	94	1.7	Feb 13, 2017
	Egypt	2017–2018	AFP patient	3	1	0	0	2.1	94	1.9	Feb 13, 2017
	Iran	2017	Non-AFP PID	3	0	1	0	1.3	99	1.1	May 18, 2017
	Iran	2015–2017	Non-AFP PID	2	0	1	0	4.1	99	3.7	Mar 13, 2017
	Israel	2016–2017	Non-AFP SCID	2	0	1	0	1.8	98	1.6	Jun 3, 2017
	South Africa	2017–2018	AFP patient	3	1	0	0	2.0	66	1.8	Jun 29, 2018
	Tunisia	2016–2017	AFP patient XLA	3	1	0	0	1.2	98	1.1	Jan 11, 2017
	United Kingdom	2015–2017	Non-AFP PID	2	0	1	0	17.94	94	>30	May 11, 2017
	West Bank and Gaza Strip	2016–2017	Non-AFP SCID	2	0	1	0	1.0	94	0.9	Feb 8, 2017
**Total iVDPV**	**—^¶¶^**	**—^¶¶^**	**—^¶¶^**	**—^¶¶^**	**8**	**6**	**0**	**—^¶¶^**	**—^¶¶^**	**—^¶¶^**	**—^¶¶^**
**aVDPV**	Australia	2017	Environmental sample	2	0	0	1	8.4	95	7.2	Nov 21, 2017
	China	2017	AFP patient	3	1	0	0	1.1	99	1.0	Feb 14, 2017
	China	2018	Environmental sample	3	0	0	1	1.4	99	1.3	Apr 18, 2018
	China	2018	Environmental sample	3	0	0	1	1.2	99	1.1	Feb 7, 2018
	DRC	2017	AFP patient	1	1	0	0	2.7	79	2.5	Apr 1, 2017
	DRC	2017	AFP patient	2	2	0	0	1.1–1.9	79	1.0–1.8	Dec 29, 2017
	India	2017	Environmental sample	2	0	0	1	1.2	88	1.1	Mar 29, 2017
	India	2018	Environmental sample	3	0	0	1	1.1	88	1.0	May 16, 2018
	Nigeria	2017	Non-AFP	2	0	1	0	0.7	40	0.7	Mar 2, 2017
	Nigeria	2017	Environmental sample	2	0	0	12	0.7–1.1	40	0.6–1.0	Apr 17, 2017
	Pakistan	2017	Environmental sample	2	0	0	5	0.7–0.8	75	0.6–0.7	Jul 15, 2017
	Somalia	2018	Environmental sample	2	0	0	1	0.7	47	0.6	Mar 1, 2018
**Total aVDPV**	**—^¶¶^**	**—^¶¶^**	**—^¶¶^**	**—^¶¶^**	**4**	**1**	**23**	**—^¶¶^**	**—^¶¶^**	**—^¶¶^**	**—^¶¶^**

**Abbreviations:** AFP = acute flaccid paralysis; aVDPV = ambiguous VDPV; cVDPV = circulating VDPV; DRC = Democratic Republic of the Congo; IPV = inactivated poliovirus vaccine; iVDPV = immunodeficiency-associated VDPV; OPV = oral poliovirus vaccine; PID = primary immunodeficiency; SCID = severe combined immunodeficiency; XLA = X-linked agammaglobulinemia.

* Total years detected for previously reported cVDPV outbreaks (DRC and Syria).

^†^ Outbreaks list total cases clearly associated with cVDPVs. Some VDPV case isolates from outbreak periods might be listed as aVDPVs. HLO-1, MAN-1, MON-1, BAN-1, BAN-2, JIS-1, and SOS-3 indicate independent cVDPV emergences and designate the location of the emergence and the number of emergences in a particular geographic region.

^§^ Total cases for VDPV-positive specimens from AFP cases and total VDPV-positive samples for environmental (sewage) samples.

^¶^ Contacts and healthy child sampling.

** Percentage of divergence is estimated from the number of nucleotide differences in the VP1 region from the corresponding parental OPV strain.

^††^ Coverage with 3 doses of OPV; data from the 2017 World Health Organization (WHO) Vaccine Preventable Diseases Monitoring System (2017 global summary) and WHO-United Nations Children’s Fund coverage estimates, http://www.who.int/gho/immunization/poliomyelitis/en/. National data might not reflect weaknesses at subnational levels.

^§§^ Duration of cVDPV circulation was estimated from extent of VP1 nucleotide divergence from the corresponding Sabin OPV strain; duration of iVDPV replication was estimated from clinical record by assuming that exposure was from initial receipt of OPV; duration of aVDPV replication was estimated from sequence data.

^¶¶^ Not cumulative data.

## Selected cVDPV Emergences from the Reporting Period

**Democratic Republic of the Congo**. Three distinct cVDPV2 emergences (designated HLO-1, MAN-1, and MON-1, per detection location and number of emergences in a geographic region) were detected during February 2017–June 2018. The HLO-1 emergence circulated in Haut Katanga, Haut Lomami, Ituri, and Tanganyika provinces. The MAN-1 and MON-1 emergences had limited circulation in Maniema and Mongala provinces, respectively. Multiple supplementary immunization rounds with mOPV2 were conducted in outbreak provinces and adjacent high-risk areas in response to the cVDPV2 emergences.

**Horn of Africa (Kenya and Somalia).** Two distinct cVDPV emergences were detected during October 2017–May 2018. A cVDPV2 emergence (designated BAN-1) was detected in three environmental sites in Mogadishu, Somalia, (Banaadir province) and in one environmental site in Nairobi, Kenya. Two cVDPV2 cases linked to BAN-1 also were detected in Gedo and Hiran provinces of Somalia. A cVDPV3 emergence (BAN-2) also was detected in the same three environmental sites; during February–May 2018, cVDPV3s associated with BAN-2 emergence were detected in three environmental sites in Mogadishu and in three cases in Middle Shabelle and Hiran provinces of Somalia. The launch of a new environmental sampling site in Mogadishu, Somalia, in October 2017 led to the immediate detection of type 2 cVDPV and subsequent detection of two type 3 cVDPVs and one aVDPV2. Outbreak response included three supplementary immunization rounds with mOPV2 in Somalia (December 2017–May 2018) and one mOPV2 round in Kenya (May 2018).

**Nigeria.** During January–June 2018, two concurrent cVDPV2 emergences were detected in Nigeria, one in the states of Gombe, Jigawa, and Yobe (designated JIS-1), and the other in Sokoto state (SOS-3). The JIS-1 emergence was detected in 18 cVDPV2 isolates from environmental samples and in isolates from four persons with AFP. Seventeen SOS-3 cVDPV2s were isolated from sewage samples collected in Sokoto. Outbreak response included two supplementary immunization rounds with mOPV2 conducted in four states (Bauchi, Gombe, Jigawa, and Sokoto) in May 2018.

**Papua New Guinea.** During April–June 2018, circulating VDPV1s were isolated from three patients with AFP and two contacts in the provinces of Easter Highlands and Morobe. Reported routine vaccine coverage at the subnational level has been low (50%) for years; bOPV outbreak response campaigns are ongoing.

## Detection of iVDPVs

During January 2017–June 2018, 14 iVDPVs were reported from nine countries, including seven type 3 iVDPVs (iVDPV3), five type 2 iVDPVs (iVDPV2), and two type 1 iVDPVs (iVDPV1). (Table). Six of the iVDPV isolates were newly detected since the last report. Since introduction of OPV in 1961, the cumulative iVDPV serotype distribution is iVDPV2 (66%), iVDPV3 (17%), iVDPV1 (12%), and heterotypic mixes (i.e., types 1 and 2 or types 2 and 3) (5%).

## Detection of aVDPVs

During January 2017–June 2018, the number of countries with detected aVDPVs decreased to seven from 11 described in the previous report (*3*) (Table). Among 28 detected aVDPVs, 23 were type 2 (aVDPV2) (predominantly after mOPV2 responses to cVDPV outbreaks), four were type 3 (aVDPV3), and one was type 1 (aVDPV1); 23 (82%) aVDPVs were isolated from environmental samples. Detection of aVDPVs in settings with <60% polio vaccination coverage might indicate a risk for cVDPV emergence and further spread. A highly divergent aVDPV2 (8.4% VP1 divergence) was isolated from an environmental sample collected in metropolitan Melbourne, Australia on November 21, 2017. This environmental isolate had genomic sequence characteristics compatible with iVDPVs.

## Discussion

During January 2017–June 2018, the number of reported cVDPV outbreaks and the total number of reported cVDPV cases in these outbreaks increased from the January 2016–June 2017 reporting period (*3*); new cVDPVs were detected in DRC, Kenya, Nigeria, Papua New Guinea, and Somalia during the January 2017–June 2018 reporting period. Cases have continued to be identified in 2018 after this reporting period in DRC, Nigeria, Papua New Guinea, and Somalia.

The continued expansion of sewage sampling (244 sites in 41 countries) (*8*) has increased the frequency of detection of WPV, VDPV, and residual PV2 excretion after mOPV2 vaccination during outbreak responses. As of April 2018, the number of environmental sites in countries with recent active WPV transmission (Afghanistan, Nigeria, and Pakistan) increased from 21 at the end of 2011 to 153. Partially as a result of supplemental surveillance measures for VDPVs among patients with primary immunodeficiencies (*9*), the number of known iVDPV excretors has increased; the antiviral pocapavir has recently been used to treat iVDPV excretors under compassionate use protocols in three countries (Task Force for Global Health, personal communication, October 2018).

Gaps in immunity to poliovirus type 2 remain in high-risk areas and continue to increase with time after the 2016 tOPV-to-bOPV switch. Continued detection of VDPV2s in 2017 and into 2018 underscores the existence of substantial populations of children who missed PV2 immunization before the switch. Some cVDPV2 detections occurred in or near security-compromised areas (DRC, Somalia, and Syria), where surveillance quality is uncertain. Likewise, new type 1 and type 3 cVDPV outbreaks highlight the importance of maintaining high levels of poliovirus immunity against these serotypes, as well as sensitive AFP surveillance. During the reporting period, VDPV cases outnumbered WPV cases; however, documentation of eradication of WPV is required before global OPV use can cease. Cessation of all OPV use after certification of polio eradication will eliminate the risk for VDPV emergence.

**Summary**

**What is already known about this topic?**

Vaccine-derived polioviruses (VDPVs) can circulate in settings of low population immunity or during outbreaks.

**What is added by this report?**

After the 2016 synchronized switch from trivalent oral poliovirus vaccine (OPV) (types 1, 2, and 3) to bivalent OPV (types 1 and 3), transmission of type 2 circulating VDPVs (cVDPVs) was detected in countries with subpopulations of children who missed immunization to type 2 poliovirus before the switch. Types 1 and 3 cVDPVs were identified in Papua New Guinea and Somalia, respectively.

**What are the implications for public health practice?**

All countries must maintain high population immunity to polio. Cessation of all OPV use after certification of polio eradication will eliminate the risk for VDPV emergence.
